# Immune thrombocytopenia in Kabuki syndrome, a comparison with non-Kabuki cases in the UK paediatric ITP registry

**DOI:** 10.1186/s13023-025-03743-y

**Published:** 2025-05-26

**Authors:** Lianna Reynolds, Benjamin Williams, Gerard Gurumurthy, John Grainger

**Affiliations:** 1 Royal Manchester Children’s Hospital, Manchester, UK; 2 Greater Manchester Mental Health, Manchester, UK; 3 School of Medical Sciences, University of Manchester, Manchester, UK

**Keywords:** Kabuki, Thrombocytopenia, ITP, Genetics

## Abstract

**Background:**

This study aims to compare the clinical presentation of Immune Thrombocytopenia (ITP) in children with Kabuki syndrome (KS) to those with sporadic ITP in the UK Paediatric ITP Registry. The Margot et al. analysis of a Kabuki database identified that children with KS had higher rates of chronic ITP and of other haematological abnormalities. This study aims to identify if children with KS do exhibit these features compared to the sporadic ITP population using data from the UK Paediatric ITP Registry between January 2006 and February 2020.

**Results:**

Of 2013 ITP patients, five had a confirmed diagnosis of KS, representing a 0.25% prevalence (95% CI = 0.031 – 0.47%). The relative prevalence of ITP in KS was estimated at 79 (95% CI = 10–149, *p* < 0.0001). Clinical presentations were similar between KS and non-KS children, with non-significant differences in severity of bleeding and platelet counts. One KS patient exhibited chronic ITP and another presented with symptoms not exclusively attributable to thrombocytopenia.

**Conclusions:**

Our findings suggest that the clinical presentation and course of ITP in children with KS are comparable to those of general ITP patients. Despite the elevated risk of ITP in KS, the manifestations of the condition do not differ significantly.

## Background

Kabuki syndrome (KS) is a rare congenital regulopathy, characterised by congenital anomalies and dysfunction in multiple organ systems, with an estimated population prevalence of 1 in 32,000 [1, 2]. Although no consensus diagnostic criteria exist, clinical features include characteristic facies, skeletal abnormalities, intellectual disability, short stature and visceral malformations. It includes endocrine, immunological and haematological disorders [1, 3]. One of the haematological disorders linked to KS is immune thrombocytopenia, with the largest study to date finding a prevalence of 7.3% [4–6]. 

The precise mechanism by which the genetic mutations which cause KS might lead to an increased risk of ITP is not known. However, the genes mutated in KS type 1 (lysine- specific methyltransferase 2D; *KMT2D*) and KS type 2 (lysine-specific demethylase 6 A; *KDM6A*) have widespread roles in epigenetic regulation of transcription factors [7–10]. This includes pathways important for maturation and proliferation in a range of B- (*KMT2D*) and T-cell (*KDM6A*) lineages, including follicular T-helper cells [10, 11]. It is thought that auto-reactive follicular T-helper cells presenting self-antigens from splenic macrophages to autoreactive B cells is the causal chain of immunological events which cause ITP [12]. This presents a putative immunological causal link between KS and ITP.

In the analysis of the French KS registry by Margot et al., 12 out of 13 patients with KS and ITP had chronic ITP [6]. Overall, there is little data on how ITP in KS may differ from sporadic cases of ITP. We identified five cases of ITP with known comorbid Kabuki syndrome in the UK Paediatric ITP Registry. We sought to compare the clinical presentation of children with KS to a cohort of children with sporadic ITP. Through analysing a different dataset we aimed to identify if children with ITP and KS do indeed have longer duration of illness and a higher rate of other haematological abnormalities when compared to the sporadic ITP patient group.

## Methods

We used data from the national UK Paediatric ITP registry, a multi-centre prospective clinical registry of new cases of ITP between January 2006 and February 2020 [13]. Registry data is entered within 12 months of initial diagnosis for all children aged between 2 months and 16 years, either directly by the patient’s clinical team or over the phone with the database data manager. Follow up data is requested six months after registration and actively sought to reduce non-response. No further information is collected from children whose ITP has resolved, whereas data is collected annually on children with ongoing ITP. All data is electronically stored in an anonymised form on a secure national database. The study was approved by the Northern and Yorkshire research ethics committee.

We performed a descriptive analysis of the five Kabuki cases. We then compared a range of key clinical and demographic variables using bivariate statistics. For continuous variables we used t-tests not assuming equal variances and for categorical variables we used Fisher’s exact test.

## Results

In the registry, five out of 2013 patients had a known diagnosis of KS. This gives an estimated prevalence of 0.25% (95% CI = 0.031 – 0.47%) of all ITP patients in the UK. There is no population estimate of KS in the UK but compared to the existing population estimates for KS of 1 in 32,000 this gives a relative prevalence for ITP in KS of 79 (95% CI = 10–149; *p* < 0.0001).

**Fig. 1:**
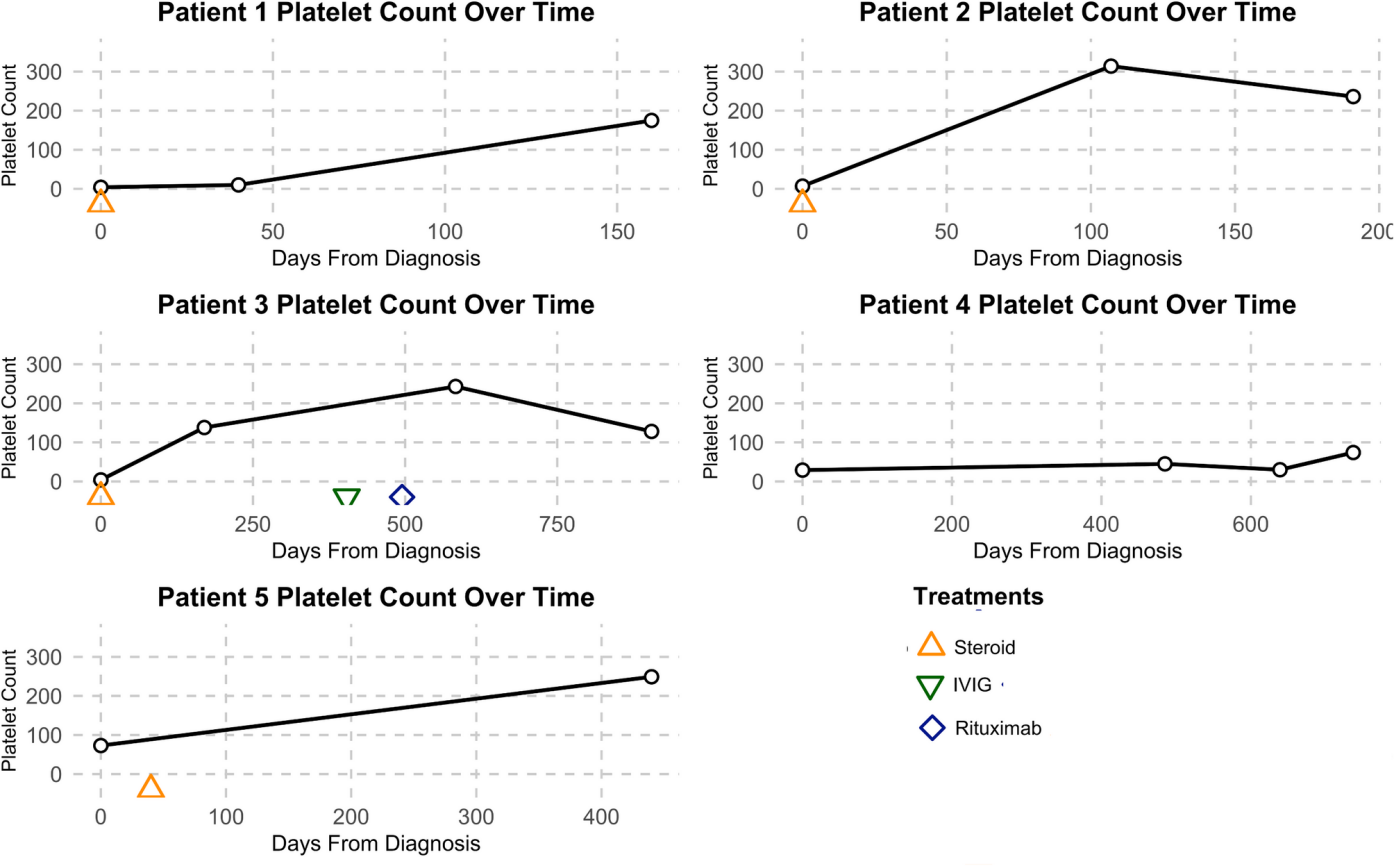
Platelet count response to interventions for each patient

Clinical and demographic data on each of these 5 children can be seen in Table 1 and trend in platelet count and treatments given in Fig. 1. Of note, Patient 4 also had a neutropenia of 0.8 (unit) at presentation however this rapidly resolved without intervention. Patient 4 was the only patient who showed persistent/chronic ITP. They had a platelet count of 64 at their final follow up, which was 291 days after initial presentation. They were subsequently lost to follow up. Patient 5 re-presented in 2024 with autoimmune haemolytic anaemia (AIHA). Patient 5 also had a history of thrombocytopenia prior to this presentation with ITP.

**Table 1:** Clinical and demographic features of identified cases of ITP in children with KS

Pt	Gender	Presentation age	Consanguinity	Vaccination	Viral illness	Atypical features	Plts	Hb	WCC	Presenting bleed severity	Bleeding site	Treatment received	Recovery by 6 months	BMA	Hospital admission at presentation
1	Girl	15.6	No	No	No	No	5	14.2	8.2	Mild	Cutaneous	Steroids only	Yes	No	No
2	Boy	8.3	No	No	Yes	No	8	13.7	4.8	Mild	Cutaneous	Steroids only	Yes	No	No
3	Boy	7.9	No	No	No	No	6	-	-	Mild	Cutaneous	Steroid, IVIG and Rituximab	Yes	No	Yes
4	Boy	8.1	No	No	No	No	29	12.3	3.8	Mild	Cutaneous		No	No	No
5	Girl	1.5	No	Unknown	No	No	73	8.9	7.1	Moderate	Cutaneous, upper GI, lower GI	IVIG only	Unknown	No	No

Treatment was given to 4 patients, as described in Table 1. This consisted of steroids for three patients, IVIG for 2 patients and Rituximab for one patient. The platelet trend and treatments received are summarised in Fig. 1. Notably, Patient 3 received Steroid treatment and Rituximab following resolution of platelet count. Unfortunately, data to identify a reason for this is unavailable, as is information regarding dose and duration of treatment. Patient 1, 2 and 3 received treatment at presentation despite only cutaneous symptoms being reported. Patient 5 presented with anaemia and both upper and lower GI mucosal bleeds, despite a relatively high platelet count of 73. This suggests their bleeding may have been related to a cause other than ITP. Patient 5 received IVIG only. Unfortunately, as a registry study we are not able to clarify the reasons for these discrepancies.

There is a relatively equal gender split, with three boys and two girls. The age at presentation was a mean of 8 years, ranging from 18 months to 15 years. This is higher than the general ITP cohort, with a mean of 5 years ranging from 2 months to 17 years and 4 months (*p* = 0.3).

Presentation was similar for those with and without KS, as seen in Table 2. There are similar rates of parental consanguinity, atypical features at presentation, viral illness preceding presentation and of vaccination prior to presentation. Platelet count was non-significantly lower in those with KS. 4 out of 5 children with KS presented with mild severity bleeding, which was not significantly different from the general ITP sample (*p* = 0.677). History of previous thrombocytopenia was non-significantly higher in those with KS. Haemoglobin was similar, however a lower white cell count in children with KS was a statistically significant difference (*p* < 0.05). Of those with KS, 75% (3 out of 4) had recovered at 6 months and 1 was lost to follow-up, this was not significantly different to the 83% 6 month recovery rate seen in the general ITP cohort.

**Table 2:** Comparison of clinical and demographic features with children with KS and ITP with children with sporadic ITP

	KS	Non-KS	Bivariate test for a difference
Age at presentation (mean)	8.3	5.7	*p* = 0.30
Thrombocytopenia Hx	0.2	0.04	*p* = 0.169
Parental Consanguinity	0%	1%	*p* = 0.94
Vaccination	0%	13%	*p* = 0.57
Viral Illness	20%	55%	*p* = 0.14
Atypical features	0%	4%	*p* = 0.82
Platelet count	13.5	24.2	*p* = 0.46
Haemoglobin	12.3	12.1	*p* = 0.86
White cell count	6.0	9.2	*p* = 0.048
Recovery by 6 months	75%	83%	*p* = 0.62

## Discussion

Our analysis provides further evidence for a substantially elevated risk of ITP in children with KS [4]. In contrast to previous cases reported in the literature, the cases we identified of ITP in KS were not more likely to be chronic [6]. Age of onset was non-significantly older amongst the children with KS, consistent with Margot et al.’s finding of greater risk in later childhood [6]. The only distinguishing feature in clinical presentation was a lower mean WCC. Immune neutropenia is documented in KS and one of the children in our cohort was neutropenic. This is of uncertain clinical significance for either diagnosis or management in our cohort [14]. Additional observation of AIHA among the KS cohort indicate a possible broader spectrum of autoimmune haematological diseases associated with KS, once again in keeping with the findings of Margot et al.. This supports the theorised underlying mechanism in KS that predisposes these patients to a wider range of autoimmune conditions, not limited to ITP.

Our study is the first to our knowledge to compare the clinical presentation of children with ITP in KS to a general cohort of children with ITP, thus clarifying the comparison to those presenting with “typical ITP” and therefore adding to the literature on this rare condition. The most striking contrast with the work of Margot et al. is the difference in rates of chronicity. In the 13 cases they identified amongst their cohort of French ITP patients 92% have chronic ITP [6]. It is difficult to explain this marked discrepancy. Both samples are small and chance may play a substantial role. The French Kabuki Syndrome network recruited from specialist clinical genetics services, meaning that patients with a chronic course may have been easier to recruit. The UK Paediatric ITP Database recruits primarily from general paediatric hospitals, and therefore may be more reflective of the general population with ITP.

We see this as a strength of the resource, and is what has enabled recruitment of a sufficient number of patients to allow identification of the cases of children with KS. It provides a strong basis for direct comparison with a paediatric ITP population seen in general paediatric settings. The statistical power and sophistication of the comparisons we can make are limited by the rarity of ITP. Given the unfamiliarity of many paediatricians with KS, it is possible that some patients with KS in the registry may be undiagnosed.

## Conclusion

Our findings suggest that, despite the elevated risk of ITP in KS, the clinical presentation and course of ITP in KS is not distinguishable from that of sporadic ITP. We hope our findings provide data to clinicians, patients and young people with KS who develop ITP.

## Data Availability

All data generated or analysed during this study are included in this published article and the UK Paediatric ITP Registry. Accessible from: https://www.uk-itp.org/.

## Abbreviations

AIHA: autoimmune haemaolytic anaemia
ITP: Immune thrombocytopenia
KDM6A: lysine-specific demethylase 6 A
KMT2D: lysine-specific methyltransferase 2D
KS: Kabuki Syndrome
